# Inflammatory Stress on Autophagy in Peripheral Blood Mononuclear Cells from Patients with Alzheimer's Disease during 24 Months of Follow-Up

**DOI:** 10.1371/journal.pone.0138326

**Published:** 2015-09-22

**Authors:** Arnaud François, Adrien Julian, Stéphanie Ragot, Emilie Dugast, Ludovic Blanchard, Sonia Brishoual, Damien Chassaing, Guylène Page, Marc Paccalin

**Affiliations:** 1 EA3808 Molecular Targets and Therapeutics of Alzheimer’s Disease, University of Poitiers, Poitiers, France; 2 Neurology Department, Poitiers University Hospital, Poitiers, France; 3 Centre Mémoire de Ressources et de Recherche, Poitiers University Hospital, Poitiers, France; 4 Geriatrics Department, Poitiers University Hospital, Poitiers, France; 5 CIC-P 1402, Poitiers University Hospital, Poitiers, France; School of Medicine, University of Belgrade, SERBIA

## Abstract

Recent findings indicate that microglia in Alzheimer’s disease (AD) is senescent whereas peripheral blood mononuclear cells (PBMCs) could infiltrate the brain to phagocyte amyloid deposits. However, the molecular mechanisms involved in the amyloid peptide clearance remain unknown. Autophagy is a physiological degradation of proteins and organelles and can be controlled by pro-inflammatory cytokines. The purpose of this study was to evaluate the impact of inflammation on autophagy in PBMCs from AD patients at baseline, 12 and 24 months of follow-up. Furthermore, PBMCs from healthy patients were also included and treated with 20 μM amyloid peptide 1–42 to mimic AD environment. For each patient, PBMCs were stimulated with the mitogenic factor, phytohaemagglutin (PHA), and treated with either 1 μM C16 as an anti-inflammatory drug or its vehicle. Autophagic markers (Beclin-1, p62/sequestosome 1 and microtubule-associated protein-light chain 3: LC3) were quantified by western blot and cytokines (Interleukin (IL)-1β, Tumor necrosis Factor (TNF)-α and IL-6) by Luminex X-MAP^®^ technology. Beclin-1 and TNF-α levels were inversely correlated in AD PBMCs at 12 months post-inclusion. In addition, Beclin-1 and p62 increased in the low inflammatory environment induced by C16. Only LC3-I levels were inversely correlated with cognitive decline at baseline. For the first time, this study describes longitudinal changes in autophagic markers in PBMCs of AD patients under an inflammatory environment. Inflammation would induce autophagy in the PBMCs of AD patients while an anti-inflammatory environment could inhibit their autophagic response. However, this positive response could be altered in a highly aggressive environment.

## Introduction

Microglia represents the immunological effector cells in the central nervous system (CNS) that continuously survey the cellular environment in the brain parenchyma [1, 2]. Once activated, microglia mainly operates as scavenger cells, producing a wide spectrum of molecules that are essential for the clearance of invading pathogens and toxic factors [such as the aggregated misfolded proteins found in Alzheimer’s disease (AD)] and for tissue homeostasis, repair and renewal [1, 2]. However, this neuroprotective role in AD might depend on intrinsic or extrinsic age-related changes [microenvironment, dysfunction of blood brain barrier (BBB)]. Indeed, primary microglia from adult mice is unable to phagocytose fibrillar amyloid peptide (Aβ) compared to microglia from early postnatal mice and this phagocytic activity seems to be inhibited by some cytokines or extracellular matrix proteins that increase with advancing aging [3, 4]. Observation of the cell morphology showed that dystrophic microglia colocalize with degenerating neuronal structures and precede the spread of tau pathology in AD brains [5]. Furthermore, the transplantation of bone marrow-derived mesenchymal stem cells can modulate immune/inflammatory responses in AD mice and improves the cognitive decline associated with Aβ deposits [6]. Neither the amyloid plaque formation and maintenance nor the amyloid-associated neuritic dystrophy depends on the presence of microglia as demonstrated in two different transgenic models of AD crossed with mice expressing an inducible suicide gene, leading to the depletion of resident microglia [7].

The contribution of blood-derived cells in the progression of AD pathology has recently evoked a lot of attention. Considering that most patients with AD have a history of cerebrovascular dysfunctions, or even periodical/chronic ischemic insults, it can be assumed that blood-derived cells can gain access to the brain of patients. This is also supported by reports indicating that 40–60% of AD patients have a leaky BBB [8]. Furthermore, many studies have reported that circulating immune cells including PBMCs can reach CNS through the BBB as part of normal immune surveillance [9]. In AD patients, activated T cells are present in both the systemic circulation and the brain [10, 11], indicating an exchange between the periphery and the CNS. By using APP/IFN-γ model of AD, authors showed that immunization with Aβ resulted in the accumulation of T cells at Aβ plaques in the brain. These T cells induced almost a complete clearance of Aβ [12]. Furthermore, bone marrow-derived microglia plays a critical role in restricting senile plaque formation in AD [13]. However, the benefit provided by these cells is still debatable. Indeed, the bone marrow-derived cell recruitment is a marginal effect in normal physiology [13], but greater in pathological conditions affecting the integrity of the CNS, such as stroke [14] and amyotrophic lateral sclerosis [15].

The molecular mechanisms that could explain the clearance of Aβ by infiltrating monocytes are poorly studied. Some mechanisms emphasized the crucial role of the expression of the chemokine receptor CCR2 to promote the monocyte infiltration across the BBB [16], others showed that microglial acidification was impaired compared to peripheral monocytes [17] and IL-1β represented also a good inducer to decrease the amyloid burden by peripheral immune cells [18]. However, the impact of an inflammatory environment in the autophagic state of PBMCs has never been studied. Yet we know that AD is characterized by an accumulation of autophagic vesicles (AVs) in dystrophic neurites [19] and recent *in vitro* study showed particular sensitivity of microglial autophagy towards an inflammatory stress [20]. Autophagy can be separated into three major distinct autophagic processes: macroautophagy, microautophagy and chaperone mediated autophagy (CMA), according to the mechanism that is used to deliver cellular substrates to the lysosomes. Macroautophagy (hereafter termed autophagy) is a lysosomal degradation pathway for long-life proteins and organelles sequestered by double membrane vesicles called autophagosomes, playing a role in metabolic homeostasis, in cell defense against many infections and degenerative states and influencing cellular immune responses [21–23]. In contrast, microautophagy is a process that results in the cytoplasm being directly engulfed at the lysosomal surface, without the involvement of intermediate transport vesicles. CMA is quite distinct from macroautophagy and microautophagy processes, in terms of selectivity and mechanism of degradation. CMA is responsible for the selective degradation of 30% of all soluble cytosolic proteins harboring a unique pentapeptide motif (KFERQ). This amino acid sequence is specifically recognized by a cytosolic chaperone, heat-shock cognate 70 (hsc70) and its co-chaperones, which selectively target these proteins to lysosomes for degradation [24].

Therefore, the purpose of this study is to evaluate the impact of inflammation on autophagy in PBMCs from AD patients at baseline, 12 and 24 months of follow-up. Furthermore, PBMCs from healthy patients were also included and treated with 20 μM amyloid peptide 1–42 to mimic AD environment. Phytohaemagglutin (PHA)-stimulated PBMCs were cultured with or without the compound C16 well known as double stranded-RNA protein kinase (PKR) inhibitor and anti-inflammatory drug [25, 26] in order to find whether an inflammatory environment could modulate autophagy. To evaluate the inflammatory response, we focused only on three major cytokines IL-1β, TNF-α and IL-6 largely described in AD [27–29] and sensitive to the anti-inflammatory drug C16 as previously published [26]. Results showed that inflammation would induce macroautophagy in the PBMCs while an anti-inflammatory environment could inhibit macroautophagic response. However, this positive autophagic activity of AD PBMCs could be altered in a very aggressive amyloid environment.

## Materials and Methods

### Suppliers of chemical products

Ficoll Histopaque^®^-1077, new born calf serum, phytohaemagglutin (PHA), sterile dimethylsulfoxide (DMSO) HybriMax^®^, the β-amyloid peptide (Aβ42), sodium fluoride (NaF), phenylmethylsulfonyl fluoride (PMSF), triton X-100, protease and phosphatase inhibitor cocktails and all reagent-grade chemicals for buffers were obtained from Sigma (St Quentin Fallavier, France). RPMI 1640 medium, 200 mM L-Glutamine, 5,000 units of penicillin (base) and 5,000 of streptomycin (base)/ml mixture (PS), Quant-it protein assay, Novex^®^ 4–20% Tris-Glycine gels, NuPAGE^®^ 4X LDS sample buffer, NuPAGE^®^ sample reducing agent (10X), Novex^®^ Tris-Glycine SDS running buffer and NuPAGE^®^ antioxidant, iBlot^®^ transfer stack regular (Nitrocellulose) from Gibco-Invitrogen (Fisher Bioblock Scientific distributor, Illkirch, France); the imidazolooxindole compound C16 from Merck Chemicals Calbiochem^®^ (Merck Millipore, Billerica, Massachusetts, USA). Primary antibodies and secondary anti-rabbit G immunoglobunins (IgG) antibody conjugated with horseradish peroxydase (HRP) from Fisher Scientific (Illkirch, France) except microtubule-associated protein-light chain 3 (LC3) and p62/sequestosome 1 (SQSTM1) from MBL (Clinisciences distributor, Nanterre France), anti-β actin from Sigma (St Quentin Fallavier, France), Bovine Serum Albumine IgG free from Jackson ImmunoResearch (Interchim distributor, Montluçon, France). X-MAP^®^ luminex Kit for cytokine assay from Merck Millipore (St-Quentin-en-Yvelines, France).

### Clinical study

Patient were selected from an ancillary study to a clinical research project (CYTOCOGMA), investigating the predictive value of peripheral cytokine and chemokine levels at diagnosis on the cognitive decline in patients with AD through a 2-year long follow-up. Patients were selected in the memory center (Centre Mémoire de Ressources et de Recherche) between November 2010 and December 2012. The diagnosis of AD was established according to the National Institute of Neurological and Communicative Disorders and the Stroke Alzheimer’s Disease and Related Disorders Association (NINCDS-ADRDA) criteria. Inclusion criteria were as follows: Mini Mental-State Examination (MMSE) scores from 16 to 25, and patient naive of symptomatic treatment for AD (cholinesterase inhibitors and memantine). Exclusion criteria were: inflammatory disease, chronic or acute infection, and neoplasia, treatment with non-steroid anti-inflammatory drug or corticosteroids and C-reactive protein (CRP) level > 10 mg/L.

The institutional review board (‘comité de protection des personnes Ouest 3’) approved the study, and patients signed a written informed consent. The study was declared on ClinicalTrial.gov.NCT01351142.

Cognitive evaluation included MMSE and ADAScog scale (Alzheimer's Disease Assessment Scale-cognitive subscale). Biological assessment included CRP, inflammatory response (IL-1β, IL-6 and TNF-α) and autophagy monitoring in PHA-stimulated PBMCs at day of inclusion (baseline or D0) and after 12 (M12) and 24 (M24) months of follow-up. In this study, we choose to focus on monitoring the functionality of autophagy in an inflammatory environment at baseline and after 12 and 24 months of follow-up. Each patient was his own control as PBMCs were treated or not with an anti-inflammatory as explained below.

As it is difficult to recruit healthy patients for a longitudinal study, we studied the functionality of autophagy and the inflammatory response of PBMCs from healthy patients cultured in different conditions as explained below.

### Extraction and culture of peripheral blood monocellular cells

All consultations were held in the morning and blood samples were sent to the laboratory for PBMCs extraction in the next hour sampling. PBMCs were isolated using Ficoll Histopaque^®^-1077 density gradient centrifugation at 2,500 rpm, at 4° C for 20 min. The PBMCs were extracted and washed by centrifugation at 1,500 rpm at 4°C for 5 min in 30 mL of sterile phosphate buffer saline (154 mM NaCl, 1.54 mM KH_2_PO_4_, 2.71 mM Na_2_HPO_4_ 7H_2_O pH 7.3) three times. PBMCs were resuspended in 1 mL complete culture medium (RPMI 1640 medium supplemented with 10% new born calf serum, 1% L-glutamine, 1% PS and 0.3 mg/mL PHA). Cells were counted using a KOVA cell with blue acetic. PBMCs were seeded at 10^6^ cells/well in 6-well plates. Cells were cultured at 37°C in 95% humidified 5% CO_2_ cell culture incubator for 48 hr.

### Chemical treatments and cellular lysis

For each AD patient, the PHA-stimulated PBMCs were treated with either 1 μM C16 (specific inhibitor of PKR with anti-inflammatory properties) or 0.8% DMSO (vehicle of C16) the day of seeding and 24h after incubation in 1 mL complete RPMI 1640 medium as described previously [26].

For healthy PBMCs, same experimental conditions were performed. To evaluate the impact of amyloid peptide in healthy PBMCs, we added other conditions. PHA-stimulated PBMCs were also incubated in serum-free RPMI 1640 medium with or without 20 μM Aβ42. As for AD PBMCs, we investigated also the treatment of 1 μM C16 or its vehicle in these conditions. Aβ42 was previously incubated 72hrs at 37°C for aggregation as recommended by supplier. This Aβ42 solution contained monomers at 4 kDa, oligomers at 8 and 12 kDa and fibrils as described by Couturier et al., 2011 [30].

After a 48h treatment, PBMCs were isolated by centrifugation at 1500 rpm during 5 min at RT, the supernatants were stored at -80°C until X-MAP^®^ luminex assay for the measure of inflammatory factors. Pellets were lysed in 150 μL of lysis buffer (50 mM Trizma^®^ base, 50 mM NaCl pH 6.8, 1% Triton X100, 1 mM PMSF, 50 mM NaF, 1% protease inhibitor cocktail and 1% phosphatase inhibitor cocktail). The lysates were sonicated (output control 2, duty cycle 25%, 5 pulsations) and centrifuged at 15,000 *g* for 15 min at 4°C. The supernatants were saved and analyzed for protein determination using Quant-it protein assay with Qubit^®^ material. All samples were frozen at −80°C until X-MAP^®^ luminex assay and immunoblotting and analyzed at the end of follow-up of 24 months to use the same batch Luminex kits and allow deposits of samples at baseline, 12 and 24 months from each patient on the same blot. For healthy PBMCs, blots grouped all conditions: serum medium with or without C16, serum-free medium with or without C16 and 20 μM Aβ42 in serum-free medium with or without C16. Same batch Luminex kits were used for healthy and AD patients.

### Immunoblotting

Samples (40 μg proteins of cell lysates) were prepared for electrophoresis by adding NuPAGE^®^ LDS 4X LDS sample buffer and NuPAGE^®^ Sample Reducing Agent (10X). Samples were then heated up 100°C for 5 min and loaded into Novex^®^ 4–20% Tris-Glycine mini gels, run at 125 V for 150 min in Novex^®^ Tris Glycine SDS running buffer containing NuPAGE^®^ antioxidant. Gels were transferred to nitrocellulose membranes using the iBlot^®^ Dry blotting system set to program 20V for 7 min. Membranes were washed for 10 min in Tris-buffered saline/Tween (TBST: 20 mM Tris-HCl, 150 mM NaCl, pH 7.5, 0.05% Tween 20) and blocked 2 hours in TBST containing 5% bovine serum albumin (BSA). Blots were incubated with primary antibody in blocking buffer overnight at 4°C. Antibodies used were rabbit anti-Beclin-1 (1:500), rabbit anti-p62/SQSTM1 (1:500), rabbit anti-LC3 (1:500). Membranes were washed twice with TBST and then incubated with the HRP-conjugated secondary anti-rabbit antibody (1:1,000) during 1 hour at RT. Membranes were washed again and exposed to the chemiluminescence Luminata Forte western HRP substrate (Millipore, St Quentin en Yvelines, France) followed by signal capture with the Gbox system (GeneSnap software, Syngene, Ozyme distributor). After 2 washes in TBST, membranes were probed with mouse antibody against β-actin (1:100,000) overnight at 4°C. They were then washed with TBST, incubated with HRP-conjugated secondary anti-mouse antibody (1:1,000) for 1 hr, exposed to the chemiluminescence Luminata Classico western HRP substrate and signals were captured. Automatic image analysis software was supplied with Gene Tools (Syngene, Ozyme distributor). *Ratios* protein/β-actin were calculated and shown in the corresponding figures.

### X-MAP^®^ Luminex assay

Human cytokine Luminex custom 3-plex kits (for TNFα, IL1β, IL-6) were purchased from Millipore (St-Quentin-en-Yvelines, France). The assay was performed in a 96-well plate and all reagents were prepared according to the Millipore instructions. Each well was cleaned and pre-wet with 200 μL of assay buffer on plate shaker (Titrimax^®^ Fisher) at 450 rpm during 10 min at RT. Assay buffer was removed by turning the plate. Assay buffer, lysis buffer or culture medium was used as a blank, each standard from a range of concentrations (3.2 to 10,000 pg/mL), quality controls and samples were added in duplicate to the appropriate wells. The mixed magnetic microbead solution was sonicated and vortexed prior to adding 25 μL into each well. The plates were sealed and incubated with agitation on a plate shaker at 750 rpm for overnight at 4°C in a darkroom. Plate were put on the magnetic support (microbead stayed around the well) then fluid was removed by using a multichannel pipette with very thin tips for not touch the periphery where are the beads. Each well was washed twice with 200 μL of wash buffer with plate shaker at 450 rpm during 2 min at RT. 25 μL of biotinylated detection antibodies were added per well, and plate were incubated in dark room at RT on a plate shaker at 750 rpm during one hour. Then 25 μL/well streptavidin–phycoerythrin solution was added, and plates were incubated on a plate shaker at 750 rpm during 30 min at RT, protected from light. Plates were washed twice with 200 μL of wash buffer. Microbeads were resuspended in 150 μL/well of sheath fluid on plate shaker at 450 rpm for 5 min at RT. Assessment was made using luminex-200^®^ instrument and xPONENT^®^ software. An acquisition gate of between 8,000 and 15,000 was set to discriminate against any doublet events and ensure that only single microbeads were measured. 50 beads / assay were collected and median fluorescence intensities (MFIs) were measured. Sensitivity limit was 0.8, 0.9 and 0.7 for IL-1β, IL-6 and TNFα, respectively. MFIs were converted to concentrations using the equation of standard range of the appropriate cytokine using Milliplex^®^ Analyst Software. The concentration of each analyte for each sample was calculated using the best parameter logistic fit curve generated for each analyte from the seven standards using Milliplex^TM^ Analyst Software. Results were expressed as pg/mL for culture media and as pg/mg protein for PBMC lysates.

### Statistical analysis

All statistical analyses were carried out using the SAS 9.2 software package (SAS Inc, Cary, NC, USA). Inflammatory and autophagic factors are described by mean and standard error of the mean (SEM), clinical variables are summarized by mean and standard deviation (SD).

Intra group comparisons between DMSO and C16 conditions were performed using non parametric Wilcoxon matched-pairs signed rank test. Biological and clinical values measured at D0, M12 and M24 for AD patients and those measured in three experimental conditions (serum medium, serum free medium and 20μM Aβ42 in serum free medium) for healthy patients were compared by ANOVA for repeated measures followed by post-hoc Sheffe’s test if necessary. Non parametric Friedman’s test was also used.

The correlations between the levels of inflammatory factors, the rate expression of autophagy markers and cognitive scores were investigated with the Spearman correlation coefficient (rho) calculation. The level of significance was p < 0.05.

## Results

### Patient characteristics

Thirty seven patients aged 78 ± 8 years (54 to 90 years), 14 men (38%) and 23 women (62%), were included. Because of 3 deaths, 4 patients lost of follow-up and 8 patients with a CRP > 10 mg / mL, the numbers of total patients were 33 and 28 at M12 and M24, respectively. Both MMSE and ADAScog scores at 24 months were significantly different to those at baseline with a loss of almost 3 points for MMSE and a gain of around 3 points for ADAScog (Table 1). Furthermore, scores at M24 were significantly different to those at M12 both for MMSE (-1.24 points) and ADAScog (+3.28 points) (Table 1). Seventy percent of patients underwent a symptomatic treatment, usually started at the time of diagnosis. All symptomatic treatments were prescribed (acetylcholinesterase inhibitors and memantine). In 8 cases, treatment was dropped out because of side effects.

**Table 1:** MMSE and ADAScog scores from AD patients at baseline, 12 and 24 months follow-up.

	D0 (n = 37)	M12 (n = 33)	M24 (n = 28)
MMSE	20.43 ± 2.63	18.85 ± 4.41	17.61 ± 4.46*** ^†^
ADAScog	15.70 ± 5.76	15.61 ± 9.58	18.89 ± 9.42** ^††^

AD patients naive of treatment for AD and have a CRP < 10 mg/L were included in this ancillary clinical study (part of Cytocogma program). Cognitive evaluation included MMSE and ADAScog scale through a 2-year long follow-up. Mean ± SD of MMSE and ADAScog scores was indicated in the table. For intra-individual comparison between D0 and M24

**p<0.001

***p<0.0001 and between M12 and M24

^†^p<0.05

^††^p<0.01 using ANOVA repeated measures and post-hoc Sheffe’s test.

Furthermore, ten healthy patients aged 88 ± 7 years (77 to 101 years), 3 men and 7 women were included in this ancillary study.

### Inflammatory levels

The anti-inflammatory effect of the C16 drastically reduced the cytokine levels in PHA-stimulated PBMCs both in intra- and extracellular media at D0, M12 and M24 (Table 2). Intracellular and extracellular levels of IL-1β were on average 106 and 627 times lower after the C16 treatment, respectively. For TNF-α, the C16 treatment reduced intracellular levels about 10-fold and 16-fold extracellular levels. Concerning IL-6, intracellular and extracellular levels were on average 106 and 156 times lower after the C16 treatment, respectively. Moreover at M12 and M24, the levels of IL-6 in C16-treated PBMCs were 1.7-fold lower than those measured at D0. In vehicle-treated PBMCs, TNF-α levels, in both intra- and extracellular media, were significantly increased at M24 compared to M12 levels (2.1-fold and 1.74-fold for intra- and extracellular media, respectively). However, no modification of the IL-1β levels was observed through the 2-year long follow-up whatever the treatment (vehicle or C16) (Table 2).

**Table 2:** Cytokine levels in PBMCs from AD patients with follow-up at 12 and 24 months after inclusion.

	Baseline (D0) n = 37		12 months (M12) n = 33		24 months (M24) n = 28	
	DMSO	C16	DMSO	C16	DMSO	C16
IL-1β _**INTRA**_	1034.97 ± 190.79	14.17 ± 3.44****	839.49 ± 137.75	7.47 ± 1.57****	1342.43 ± 176.32****	10.09 ± 2.01****
TNF-α _**INTRA**_	720.08 ± 129.28	170.83 ± 54.03****	474.20 ± 75.69	52.11 ± 9.96****	**1004.24 ± 186.43** ^†^	125.06 ± 72.14****
IL-6 _**INTRA**_	1373.31 ± 220.73	21.65 ± 3.12****	1320.86 ± 223.38	**12.49 ± 2.19** **** ^‡‡^	1816.06 ± 279.57	**12.18 ± 2.02** **** ^‡‡^
IL-1β _**EXTRA**_	14591.00 ± 2505.06	19.72 ± 3.73****	26037.26 ± 9895.80	41.17 ± 20.34****	18375.39 ± 2138.07	35.94 ± 11.43****
TNF-α _**EXTRA**_	3723.38 ± 882.26	182.28 ± 30.94****	3062.65 ± 530.27	352.11 ± 171.17****	**5341.50 ± 721.93** ^†^	266.93 ± 61.04****
IL-6 _**EXTRA**_	11952.20 ± 1641.44	65.44 ± 7.02****	14447.09 ± 3479.16	127.92 ± 42.54****	17255.82 ± 2458.10	98.92 ± 24.80****

IL1-β, TNF-α and IL-6 levels measured by the 3-plex Luminex xMAP^®^ assay in cell lysates (INTRA) and in culture media (EXTRA) at baseline (day 0 inclusion), 12 months (M12) and 24 months (M24) after pre-treatment of PBMCs with 1 μM C16 *versus* its vehicle (DMSO). Cytokine levels were expressed in pg/mg protein for INTRA and in pg/mL for EXTRA samples. Results are mean ± SEM. Intra-individual comparisons between DMSO and C16 conditions at each time

****p<0.0001 by Wilcoxon matched-pairs signed rank test, Intra-individual comparisons between D0, M12 and M24 in DMSO or C16 conditions

^†^p<0.05 compared to DMSO-treated PBMCs at M12

^‡‡^p<0.01 compared to C16-treated PBMCs at Day 0 by ANOVA for repeated measures and post-hoc Sheffe’s test. Friedman test: p = 0.0190 and p = 0.0087 for TNF-α in INTRA and EXTRA with DMSO treatment, respectively and p = 0.0052 for IL-6 INTRA with C16 treatment.

Analysis did not find any impact of the symptomatic medications on the inflammatory responses.

As in PBMCs from AD patients, results showed that C16 significantly reduced the levels of intracellular and extracellular cytokines in PBMCs from healthy patients (about 99% for IL-1β and IL-6 and 94% for TNF-α) (Table 3). After DMSO or C16 treatment, intracellular levels of these cytokines were similar whatever the conditions of medium culture (with or without serum and with or without Aβ42). However, extracellular TNF-α and IL-6 levels released by DMSO-treated PBMCs decreased in serum free medium without or with Aβ42 (61 and 71% for TNF-α, respectively and by 45% for IL-6 in both medium conditions compared to serum medium). These TNF-α and IL-6 decreases were also observed in C16-treated PBMCs cultured in serum free medium without or with Aβ42 (TNF-α: 50% with Aβ42 and 77 and 84% for IL-6, respectively compared to serum medium). However, no difference was observed for IL-1β levels after DMSO or C16 treatment whatever the culture medium condition (Table 3).

**Table 3:** Cytokine levels in PBMCs from healthy patients in three experimental conditions.

	Serum medium		Free-serum medium		Free-serum medium with Aβ42	
	DMSO	C16	DMSO	C16	DMSO	C16
IL-1β _**INTRA**_	1068.00 ± 210.40	9.75 ± 2.87**	1301.00 ± 280.90	3.31 ± 1.42**	1491.00 ± 537.40	1.48 ± 0.38**
TNF-α _**INTRA**_	920.80 ± 176.80	39.68 ± 5.95**	691.80 ± 119.90	53.88 ± 6.44**	629.10 ± 167.40	42.84 ± 9.93**
IL-6 _**INTRA**_	2167.00 ± 474.00	10.53 ± 4.45**	4239.00 ± 776.20	12.71 ± 5.56**	5025.00 ± 1510.00	7.86 ± 4.28**
IL-1β _**EXTRA**_	32865.00 ± 9541.00	95.19 ± 34.90**	31873.00 ± 7860.00	29.64 ± 8.68**	28238.00 ± 8016.00	36.60 ± 17.69**
TNF-α _**EXTRA**_	11907.00 ± 1923.00	413.50± 58.68**	4652.00 ± 901.70^†††^	349.00 ± 60.10**	3385.00 ± 726.00^†††^	211.20 ± 41.38** ^,^ ^‡‡^ ^,^ ^#^
IL-6 _**EXTRA**_	43789.00 ± 4575.00	145.80 ± 48.07**	23833.00 ± 4530.00^†††^	32.97 ± 9.20** ^,^ ^‡‡^	23734.00 ± 6739.00^†††^	23.04 ± 5.98** ^,^ ^‡‡^

IL1-β, TNF-α and IL-6 levels measured by the 3-plex Luminex xMAP^®^ assay in cell lysates (INTRA) and in culture media (EXTRA) after pre-treatment of PBMCs with 1 μM C16 *versus* its vehicle (DMSO) in three experimental conditions during 48h: serum medium, serum-free medium and serum-free medium with 20 μM Aβ42. Cytokine levels were expressed in pg/mg protein for INTRA and in pg/mL for EXTRA samples. Results are mean ± SEM. Intra-individual comparisons between DMSO and C16 conditions at each time

**p<0.01 by Wilcoxon matched-pairs signed rank test. Intra-individual comparisons between serum medium, free-serum medium, free serum medium with 20μM Aβ42 in DMSO or C16

^†††^p<0.001 compared to DMSO-treated PBMCs in serum medium

^‡‡^p<0.01 compared to C16-treated PBMCs in serum medium

^#^p<0.05 compared to C16-treated PBMCs in serum-free medium by ANOVA for repeated measures and post-hoc Sheffe’s test.

At baseline (D0), levels of intracellular cytokines from AD patients were similar to those of healthy patients after PHA stimulation in serum culture medium. However, extracellular levels of cytokines released by PBMCs from healthy patients were increased by 55%, 68% and 72% for IL-1β, TNF-α and IL-6, respectively compared with those quantified from AD PBMCs at baseline (D0) (Tables 2 and 3).

### Autophagy monitoring

To determine whether autophagy changed in PHA-stimulated PBMCs at M12 and M24 compared to D0, Beclin-1, p62, LC3 I and LC3 II were investigated. Beclin-1 is a key component of the class III PI3K (Phosphatidylinositide 3-kinases) complex which is involved in the initiation of autophagosome formation [31]; p62 is an autophagic receptor which recognizes ubiquitinylated proteins and interacts with LC3 II at the forming autophagosome [32]; LC3 is present in free cytoplasmic form as LC3 I. When LC3 I is associated with phosphatidylethanolamine of the membrane of autophagosome (through an ubiquitin-like conjugation reaction), it produces LC3 II form, a useful marker of autophagic membranes [32].

**Fig 1:**
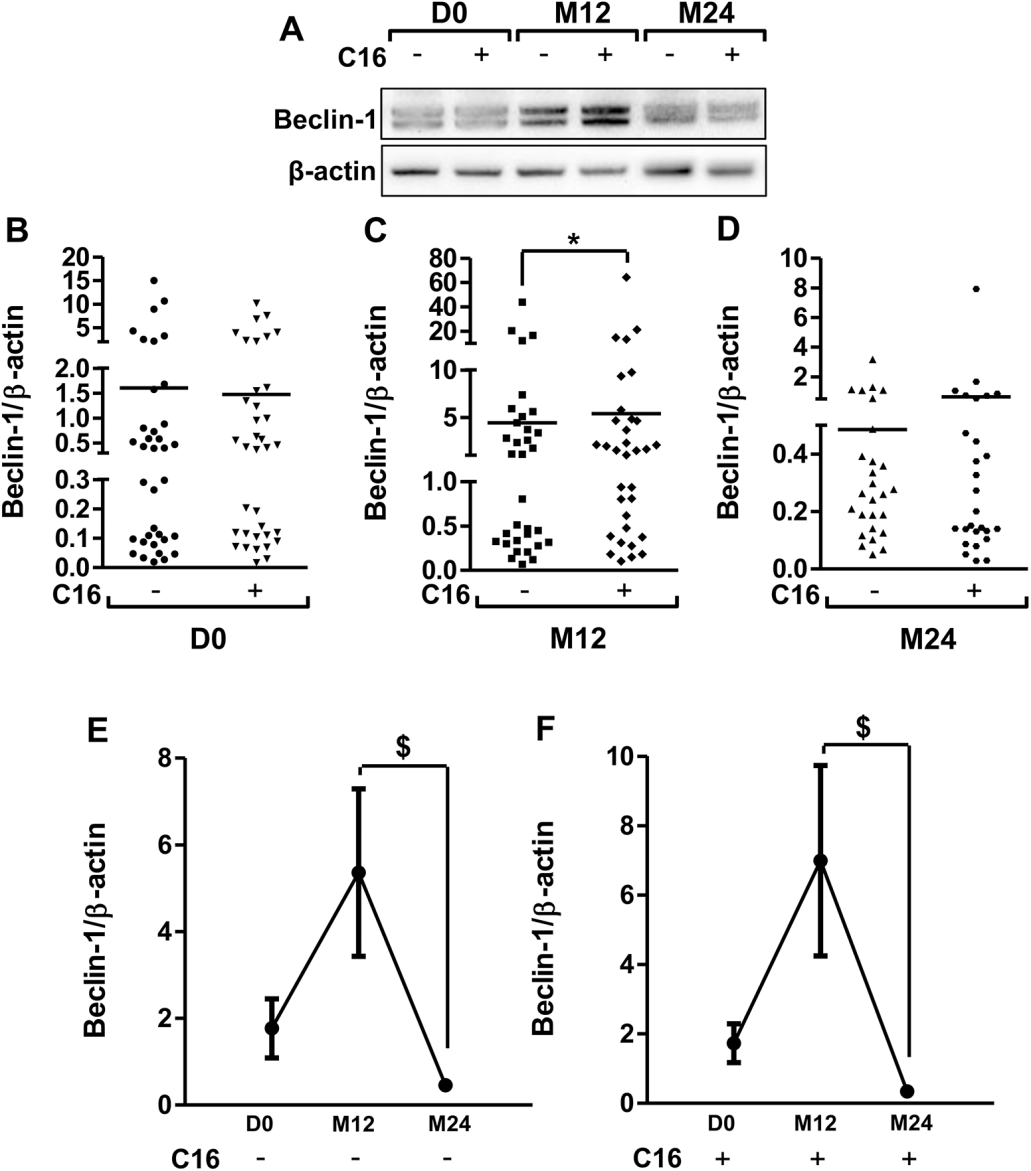
Longitudinal monitoring of Beclin-1 expression in PBMCs from AD patients. Representative immunoblots (panel A) showed the immunoreactivity of Beclin-1 in PBMCs of AD patients at the Day of inclusion (D0, panel B) and after a follow-up at 12 months (M12, panel C) and 24 months (M24, panel D). PBMCs were isolated from blood and cultured either with 1 μM C16 or its vehicle (0.8% DMSO) for 48hr as described in method section. Semi-quantitative analysis of immunoblot was performed using Gene Tools software (Syngene, Ozyme France). The immunoreactivity of protein was normalized to β-actin immunoreactivity. The line on the graphs (B to D) represents the mean of 36, 33 and 27 patients at D0, M12 and M24, respectively. The mean expression of Beclin-1 in PBMCs at D0, M12 and M24 are shown in the panels E and F, without or with the C16 treatment, respectively. *p < 0.05 compared to vehicle-treated PBMCs at M12 by Wilcoxon matched-pairs signed rank test; ^**$**^p < 0.05 compared to PBMCs at M12 by ANOVA for repeated measures and post-hoc Sheffe’s test. Friedman test: p = 0.0019 for Beclin-1 in vehicle condition and p = 0.0002 in C16 condition.

**Fig 2:**
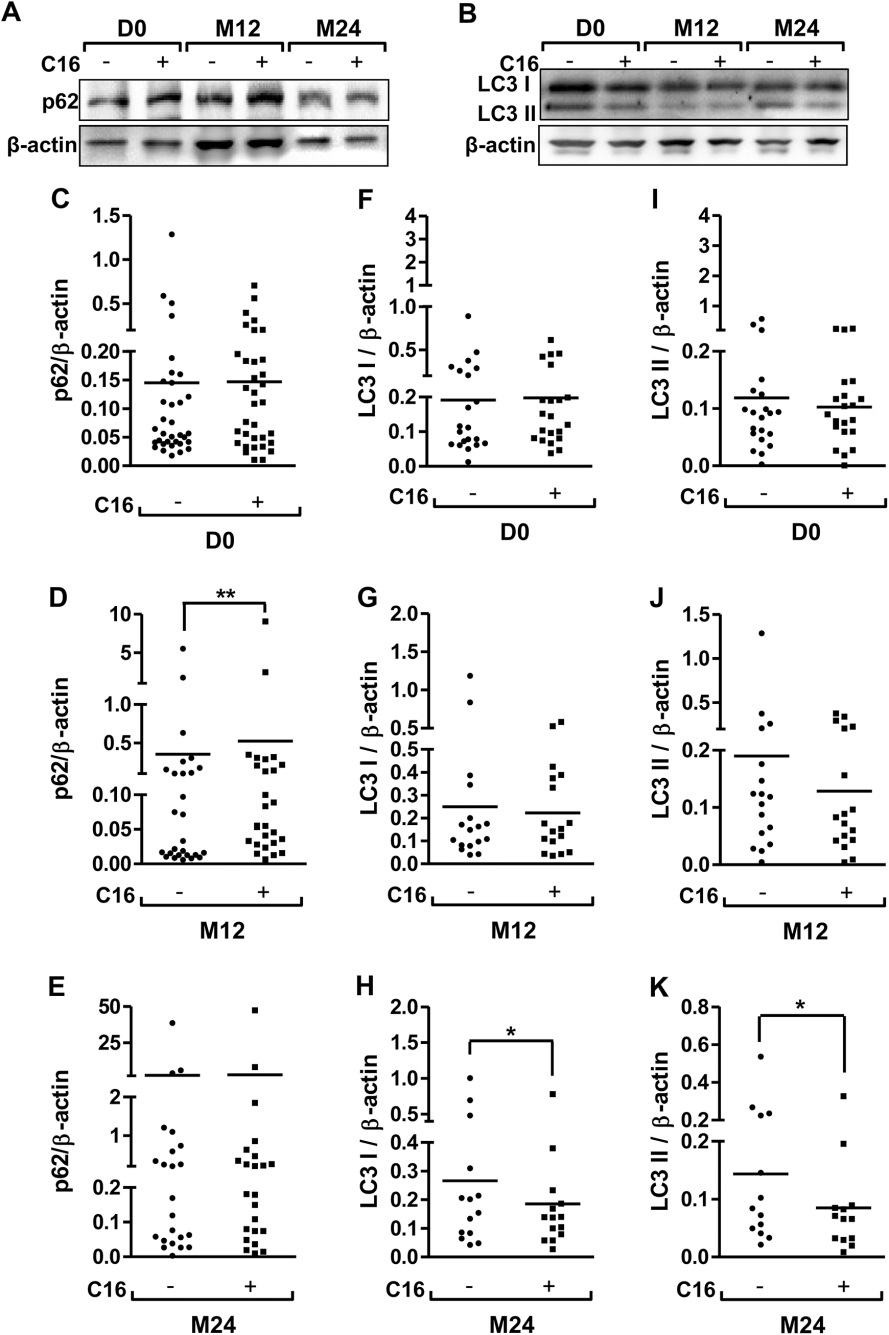
Longitudinal monitoring of p62 and LC3 expression in PBMCs from AD patients. Representative immunoblots showed the immunoreactivity of p62 (A, C, D and E), LC3 I (B, F, G and H) and LC3 II (B, I, J and K) in PBMCs of AD patients at the Day of inclusion (D0) and after a follow-up at 12 months (M12) and 24 months (M24). PBMCs were isolated from blood and cultured either with 1 μM C16 or its vehicle (0.8% DMSO) for 48hr as described in method section. Semi-quantitative analysis of immunoblot was performed using Gene Tools software (Syngene, Ozyme France). The immunoreactivity of protein was normalized to β-actin immunoreactivity. The line on the graphs represents the mean of 34, 27 and 23 patients for p62, mean of 21, 17 and 14 patients for LC3 I and mean of 21, 17 and 13 patients for LC3 II at D0, M12 and M24, respectively. Some signals were not analyzed on the blot due to a very low or absent signal. *p < 0.05, **p < 0.005 compared to respective vehicle-treated PBMCs by Wilcoxon matched-pairs signed rank test. Friedman test was not significant for these three autophagic markers.

The C16 treatment of PBMCs affected the Beclin-1 expression at M12 with a significant increase in 23% and no change at D0 and M24 (Fig 1A to Fig 1D). Between M12 and M24, Beclin-1 expression significantly decreased either with vehicle or C16 treatment (89% and 88% in DMSO and C16 conditions, respectively, Fig 1E and Fig 1F). For p62, C16 treatment induced an increase of its expression (48.5%) compared to vehicle condition at M12 but no significant changes were observed during follow-up (Fig 2A, Fig 2C, Fig 2D and Fig 2E). For LC3, a decrease in 30% and 41% for LC3 I and LC3 II, respectively was observed with C16 treatment at M24 (Fig 2B, Fig 2F to Fig 2K).

As for inflammatory responses, analysis did not find any impact of the symptomatic medications on the autophagic responses.

**Fig 3:**
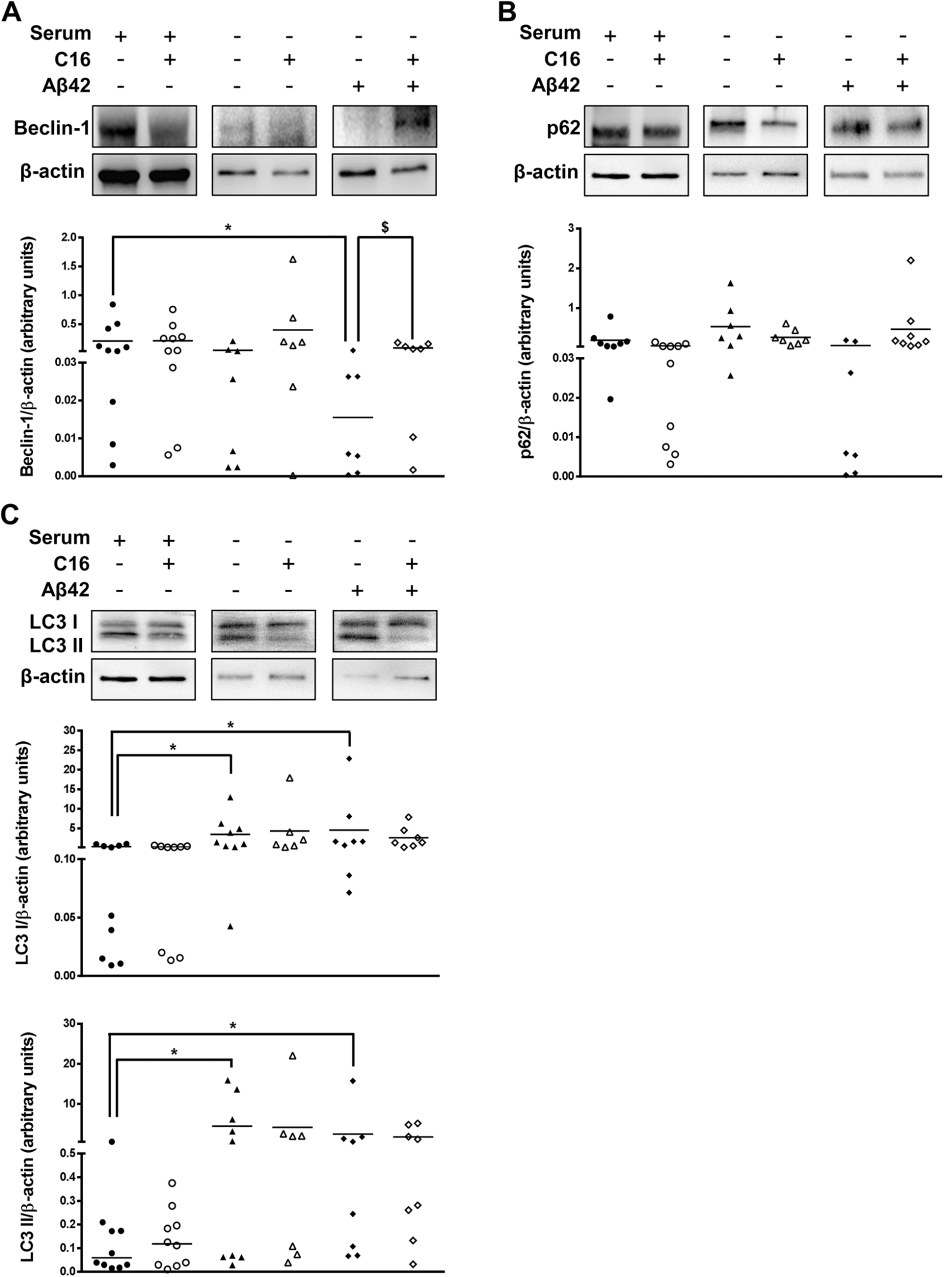
Expression of autophagic markers in PBMCs from healthy patients. Representative immunoblots showed the immunoreactivity of Beclin-1 (panel A), p62 (panel B), LC3 I and LC3 II (panel C) in PBMCs of healthy patients cultured in three experimental culture conditions (serum medium, serum free medium and 20μM Aβ42 in serum free medium) with or without 1 μM C16 or its vehicle (0.8% DMSO) for 48hrs as described in method section. Semi-quantitative analysis of immunoblot was performed using Gene Tools software (Syngene, Ozyme France). The immunoreactivity of protein was normalized to β-actin immunoreactivity. The line on each graph represents the mean. *p < 0.05 compared to DMSO-treated PBMCs in serum medium, ^$^p < 0.05 compared to Aβ42-treated PBMCs in serum free medium with DMSO by Wilcoxon matched-pairs signed rank test.

For healthy patients, in DMSO-treated PBMCs cultured in serum-free medium with 20 μM Aβ42, the Beclin-1 expression significantly decreased (92%) but was rescued by the C16 treatment (Fig 3A). No significant variation of p62 expression was observed (Fig 3B). However, LC3 I and LC3 II expressions significantly increased in DMSO-treated PBMCs cultured in serum free medium with or without 20 μM Aβ42 (Fig 3C).

### Correlation between inflammation and autophagy

Spearman correlations were performed and showed positive correlations between LC3 I or LC3 II with intracellular levels of IL-6 at D0: rho = 0.54 and 0.46 with respective p = 0.014 and 0.035 (Table 3). In addition, Beclin-1 levels were inversely correlated with intra- and extracellular levels of TNF-α at M12: rho = -0.49, p = 0.006 and rho = -0.45, p = 0.010, respectively (Table 4).

**Table 4:** Correlation between inflammation and autophagic markers in PBMCs from AD patients.

	D0			M12			M24		
	Beclin-1	LC3 I	LC3II	Beclin-1	LC3 I	LC3II	Beclin-1	LC3 I	LC3II
	(n = 37)	(n = 22)	(n = 22)	(n = 33)	(n = 17)	(n = 17)	(n = 27)	(n = 15)	(n = 14)
TNF-α	rho = -0.24	rho = 0.22	rho = 0.31	**rho = -0.49**	rho = 0.25	rho = 0.05	rho = -037	rho = 0.08	rho = 0.11
_**INTRA**_	p = 0.157	p = 0.316	p = 0.155	**p = 0.006**	p = 0.320	p = 0.830	p = 0.057	p = 0.768	p = 0.698
TNF-α	rho = 0.01	rho = 0.05	rho = 0.25	**rho = -0.45**	rho = 0.15	rho = -0.23	rho = -0.12	rho = 0.10	rho = 0.33
_**EXTRA**_	p = 0.970	p = 0.8060	p = 0.258	**p = 0.010**	p = 0.550	p = 0.350	p = 0.540	p = 0.708	p = 0.237
IL-6	rho = -0.14	**rho = 0.54**	**rho = 0.46**	rho = -0.27	rho = 0.16	rho = 0.05	rho = -014	rho = 0.26	rho = 0.29
_**INTRA**_	p = 0.412	**p = 0.014**	**p = 0.035**	p = 0.130	p = 0.510	p = 0.840	p = 0.470	p = 0.323	p = 0.300

Spearman correlations were performed between Beclin-1, p62, LC3 I and LC3 II levels and IL1-β, TNF-α and IL-6 levels measured in intra- and extracellular media (INTRA and EXTRA) of vehicle-treated PBMCs at D0, M12 or M24. No correlation was observed for p62 (data not shown). Some signals were not analyzed on the blot due to a very low or absent signal, therefore the number of patients was indicated in each row. In table, rho and p values were indicated. The level of significance was p < 0.05 and significant correlations were shown in bold.

Cognitive decline evaluated by MMSE and ADAScog scale was not correlated with any autophagy markers except LC3 I and ADAScog score at D0 (rho = -0.47 and -0.49; p = 0.03 and 0.02 in vehicle (n = 22 patients) and C16 (n = 21 patients) conditions, respectively).

For healthy DMSO-treated PBMCs, all spearman correlations between intra and extracellular cytokine levels and autophagic markers were performed and showed only two correlations: first in serum free medium with Aβ42 between extracellular TNF-α and p62 levels (rho = 0.73, p = 0.031) and second in serum-free medium between intracellular TNF-α and Beclin-1 levels (rho = 0.783, p = 0.04).

## Discussion

Recent advances show the interest of PBMCs in the repair of CNS damage following acute insults in neurodegenerative diseases [11, 12, 15]. However in AD, the mechanisms explaining the differences between PBMCs and microglia in degradation of Aβ are not fully elucidated. As AD patients displayed many AVs in dystrophic neurites, indicating a deleterious regulation of autophagy [19], it was interesting to follow the autophagic status of PBMCs which has never been explored before. In addition, we previously demonstrated in *in vitro* model of AD the importance of the relationships between autophagy and inflammation, particularly the role of the cytokine IL-1β as an autophagic inducer specifically in the microglia [20]. Recently in AD mouse APPswePS1dE9 model, we revealed two positive correlations, one between IL-1β and Beclin-1 levels and another between TNF-α and Beclin-1 levels in cortex and hippocampus, demonstrating the influence of these cytokines in the autophagic process which is failed with a great accumulation of AVs such as in AD patients [29]. The inflammatory effect on autophagy was initially reported at the periphery in various diseases including Crohn's disease [33–35], which stimulated our research to modulate the inflammatory environment of PBMCs by treating the cultures with the C16 compound, known as a specific inhibitor of PKR with anti-inflammatory properties [25, 26]. In this clinical study, we had the opportunity to monitor the impact of inflammation on autophagy PBMCs at baseline and after 12 and 24 of follow-up. Furthermore, PBMCs from healthy patients were included and exposed to amyloid peptide (20 μM Aβ42) in order to mimic AD environment.

A wide range of cytokines are overexpressed in PBMCs from AD patients in particular, TNF-α, IL1-β and IL-6 [26, 36, 37]. TNF-α and IL1-β are among the 18 signaling proteins in blood plasma that can be used to classify blinded samples from subjects with AD and control subjects with close to 90% of accuracy [38]. Many studies revealed a significant increase of IL-6 in PBMCs in patients with AD, compared to controls [39–41]. In these studies, the AD patients were at a moderate or severe stage of the disease. In our study, there was much increase of TNF-α in time (a loss of 2.82 points for MMSE between D0 and 24 months of follow-up). Comparing the cytokine levels at baseline in AD PBMCs *versus* healthy PBMCs showed on the contrary a higher extracellular cytokine response of healthy PBMCs after PHA stimulation. Other authors showed that PBMCs from age-matched healthy patients were more susceptible to inflammatory stress induced by aggregated Aβ42 (10^-4^M with serum medium) than those from AD patients [42].

In our experimental conditions, serum free medium with or without Aβ42 significantly decreased extracellular TNF-α and IL-6 levels without modifying IL-1β levels in healthy PBMCs compared to serum medium condition. In human PBMCs (without information about patients), some authors have shown that inhibition of autophagy with 3-methyl adenine (blocker of Beclin-1 complex) decreased the released TNF-α levels under inflammatory conditions while IL-1β levels increased in serum medium. This decrease of TNF-α was also observed at the transcriptional level in serum free and inflammatory medium while no modification of IL-1β mRNA levels was observed [43].

Beclin-1 was the only autophagic marker rate that changed during the patient’s follow-up. Indeed, a significant increase was observed at 12 months compared to the expression rate at 24 months of follow-up. This induction of expression is difficult to explain. However, Beclin-1 and TNF-α levels were inversely correlated at 12 months of follow-up, indicating a link with the inflammatory response of PHA-stimulated PBMCs and their autophagic process. The higher TNF-α production was correlated with the lower Beclin-1 level in PHA-stimulated PBMCs of AD patients at 12 months of follow-up. Furthermore, lower levels of Beclin-1 were measured at 24 months while TNF-α production increased. In contrast, a highly attenuated inflammatory environment induced by C16 treatment as described previously [26] led to an increase of Beclin-1 at 12 months of follow-up.

A way to explain the Beclin-1 decrease in PHA-stimulated PBMCs of AD patients may concern the proteolysis. Indeed, Beclin-1 can be cleaved by different caspases as caspase 8 known to be activated by TNF receptor (TNFR) signaling pathway [44].

Furthermore, Beclin-1 interacts with many partners to build a complex, requiring for the formation of autophagosomes as well as for the phagocytosis in microglial cells [31]. In Beclin-1 complex, some targets (Vps34, Ambra 1, Bcl-2, Bim) could be regulated by TNF-α which is a major mediator of apoptosis as well as of inflammation and immunity [45]. In these PBMCs, expression of Beclin-1, as autophagic initiator, is negatively regulated by an inflammatory environment and in particular by the TNF-α production.

Although the mechanism of inhibition is not well understood, the amyloid stress induced a reduction of expression of the Beclin-1 protein, which is capable of regulating the accumulation of amyloid peptide [46–48]. In accordance with these findings, our results showed that PBMCs from healthy patients displayed reduced expression of Beclin-1 after exposition to Aβ42. This decreased expression of Beclin-1 could disturb phagocytosis of macrophages as it was recently demonstrated a role for Beclin-mediated protein sorting at the plasma membrane in microglia [49, 50].

Besides the hypothesis about the proteolysis or its partners or amyloid peptide, Beclin-1 decrease in inflammatory environment may be due to an autophagic induction leading to consumption. To better assess autophagy, it is necessary to monitor other autophagic markers as p62 and LC3 performed in this study.

As Beclin-1, p62 increased in C16-treated PBMCs at 12 months of follow-up. The p62 protein, playing an important role in directing ubiquitinated cargos towards autophagy and degraded with cargos, contains several protein–protein interaction domains which function as scaffolds for the formation of signaling protein complexes [51, 52]. In particular, p62 can activate the NF-κB signaling pathway through its tumor necrosis factor receptor-associated factor (TRAF6) binding site [51, 52]. This NF-κB signaling pathway is known to activate autophagy. However, the compound C16 inhibits PKR which activates the NF-κB pathway as previously described in PHA-stimulated PBMCs of AD patients [26]. In addition, PKR activates autophagy [53]. It is also known that p62 can bind to Raptor a component of mammalian target of rapamycin 1 (mTORC1) and activate it [54], leading to an inhibition of autophagy. Thus, in C16 condition, the increase of p62 could be due to inhibition of autophagy through the inhibition of NF-κB, PKR signaling pathways by C16 and activation of mTOR by p62. As for Beclin-1, results of p62 indicated that PBMCs of AD patients placed in an anti-inflammatory environment could inhibit their autophagic process. However, no modification of p62 was observed in healthy PBMCs.

Concerning LC3, the total levels in mammalian cells do not necessarily change in a predictable manner, as there may be an increase in the conversion of LC3-I to LC3-II, or a decrease in LC3-II relative to LC3-I if degradation of LC3-II *via* lysosomal turnover is particularly fast. Here, results showed that levels of LC3-I and LC3-II decreased with C16 i.e. in a very low inflammatory environment after 24-months of follow-up. No modification was observed at M12 with C16 contrary to Beclin-1 and p62 which increased.

Serum starved PBMCs significantly increased the expression of the two isoforms of LC3. This increase is maintained in the presence of Aβ42 with or without C16. It is well known that autophagy is induced in serum starved cells [43, 55, 56]. In line with these observations in serum starved PBMCs (decrease of Beclin-1 and increase of both LC3 isoforms), less Beclin-1 but more LC3 protein were observed in brains of AD patients [47]. These experimental conditions could be deleterious for these PBMCs from healthy patients and induction of autophagy could lead to apoptosis [57].

At this peripheral level, autophagy in PBMCs of AD patients could be inhibited by an anti-inflammatory environment while TNF-α production could induce autophagy. Interestingly, recent results about a randomized, placebo-controlled, double-blind, phase 2 trial using subcutaneous etanercept (a TNF-α inhibitor) treatment in patients with mild to moderate AD and follow-up during 24 months were published [58]. This study showed no significant cognitive improvement by subcutaneous etanercept administration after 24 months of follow-up in AD patients [58]. This treatment might have inhibited the autophagic activity of PBMCs. At the periphery, these findings question the beneficial or deleterious role of inflammation [59, 60].

However, our data presented here are inconsistent with those obtained previously *in vitro* primary cultures of neurons/astrocytes/microglia exposed to an inflammatory stress (François et al., 2013) and *in vivo* in a transgenic mouse AD model (François et al., 2014). Indeed, inflammation leads to a blockage of autophagic flux resulting in a great accumulation of autophagic vesicles in brain as it was observed in AD patients. Furthermore, the cytokine involved is IL-1β and not TNF-α and microglia represents the most vulnerable cells to autophagy impairment. While it is evident to add further experiments to better understand the autophagy process in PBMCs, these first findings suggest that blood mononuclear cells such as monocytes, lymphocytes from AD patients could show autophagy failure in an anti-inflammatory environment while microglia would experience this phenomenon in inflammatory conditions.

As these peripheral cells were more efficient than microglia in AD [12, 61, 62], one may propose that their autophagic process would be more active for Aβ clearance than in microglia. Experiments conducted on PBMCs of healthy patients led us to make a reservation on the effectiveness of AD PBMCs *versus* microglia. Such an aggressive environment as serum free medium with high concentrations of amyloid peptide can be harmful and lead to their senescence.

While no correlation has been found for Beclin-1 and p62 with the cognitive decline evaluated by MMSE and ADAScog scales, LC3-I was inversely correlated with ADAScog score. Thus, AD patients with a high score of ADAScog at baseline have a low level of LC3-I expression, indicating an early impact on autophagy in PBMCs. This result yet not described in the literature requires further investigations to understand the link between autophagy and cognitive decline in AD.

Furthermore, all parameters of autophagy measured at 24 months of follow-up were similar to those observed at baseline and no correlation between autophagy and inflammation was noted. Patients included in this clinical study were naive of symptomatic treatment at baseline but most received such treatment after AD diagnosis. Therefore, it would be interesting to research whether the symptomatic treatment of AD may influence the autophagic response and/or inflammatory response given the crosstalks already described in the literature. The selective acetyl- and butyrylcholinesterase inhibitors reduce *ex vivo* β-amyloid-induced activation of peripheral chemo-cytokines from AD patients [63, 64]. For memantine, results are controversial [65, 66]. Furthermore, one study revealed that galanthamine hydrochlorure decreased the level of GFP-LC3 in neuroglioma H4 cells [67]. However, in this study, no impact of AD treatments was seen on PBMC parameters. Our study cannot give any conclusion on any treatment influence. This might be due to the low number of patients and the use of 4 different drugs.

## Conclusions

This study describes the first modifications of the expression of autophagic markers in PHA-stimulated PBMCs of AD patients with a negative correlation with the Beclin-1 and TNF-α levels at 12 months follow-up. The results obtained from PBMCs of healthy patients showed that aggressive environment could inhibit this response.

It is interesting to note that in Parkinson's disease (PD) and in amyotrophic lateral sclerosis (ALS), autophagy status in PBMCs was also studied. PBMCs from PD patients displayed a reduced expression of the chaperone-mediated autophagy carrier hsc70 protein [68] and results from PBMCs from ALS patients are contradictory: No significant difference of Beclin-1, LC3 immunoreactivity and mRNA Atg12 expression was observed between patients and controls although PBMC displayed a clear macroautophagy induction following exposure to rapamycin and lithium [69], while other data showed that the expression of the early autophagy-associated gene ATG12 was increased in PBMC from SCA7 patients in correlation with disease severity [70].

In the present study, results showed that inflammation would induce macroautophagy in the PBMCs of AD patients while an anti-inflammatory environment could inhibit the macroautophagic response. This new molecular interaction in peripheral cells should be completed in assessing the specificity of this interaction in AD and adding the analysis of other pro- and anti-inflammatory and autophagic markers such as ATG12.
