# Correction: Inflammatory Stress on Autophagy in Peripheral Blood Mononuclear Cells from Patients with Alzheimer's Disease during 24 Months of Follow-Up

**DOI:** 10.1371/journal.pone.0143933

**Published:** 2016-03-15

**Authors:** Arnaud François, Adrien Julian, Stéphanie Ragot, Emilie Dugast, Ludovic Blanchard, Sonia Brishoual, Faraj Terro, Damien Chassaing, Guylène Page, Marc Paccalin

Dr. Faraj Terro was not included in the author byline. He should be listed as the seventh author and is affiliated with EA3808 Molecular Targets and Therapeutics of Alzheimer’s disease, University of Poitiers, Poitiers, France; University of Limoges, Laboratory of Histology and Biology, Faculty of Medicine, Limoges, France; Service d’histologie et de cytogénétique, Hôpital de la Mère et de l’Enfant, Limoges, France. The contributions of this author are as follows: Thinking on autophagy.
